# Great tits who remember more accurately have difficulty forgetting, but variation is not driven by environmental harshness

**DOI:** 10.1038/s41598-021-89125-3

**Published:** 2021-05-12

**Authors:** Ethan Hermer, Ben Murphy, Alexis S. Chaine, Julie Morand-Ferron

**Affiliations:** 1 University of Ottawa, Ottawa, ON Canada; 2 University of Cape Town, Cape Town, South Africa; 3 Station d’Ecologie Théorique et Expérimentale du CNRS, Moulis, France; 4 Institute for Advanced Studies in Toulouse, Toulouse School of Economics, Toulouse, France

**Keywords:** Behavioural ecology, Evolutionary ecology

## Abstract

The causes of individual variation in memory are poorly understood in wild animals. Harsh environments with sparse or rapidly changing food resources are hypothesized to favour more accurate spatial memory to allow animals to return to previously visited patches when current patches are depleted. A potential cost of more accurate spatial memory is proactive interference, where accurate memories block the formation of new memories. This relationship between spatial memory, proactive interference, and harsh environments has only been studied in scatter-hoarding animals. We compare spatial memory accuracy and proactive interference performance of non-scatter hoarding great tits (*Parus major*) from high and low elevations where harshness increases with elevation. In contrast to studies of scatter-hoarders, we did not find a significant difference between high and low elevation birds in their spatial memory accuracy or proactive interference performance. Using a variance partitioning approach, we report the first among-individual trade-off between spatial memory and proactive interference, uncovering variation in memory at the individual level where selection may act. Although we have no evidence of harsh habitats affecting spatial memory, our results suggest that if elevation produced differences in spatial memory between elevations, we could see concurrent changes in how quickly birds can forget.

## Introduction

There is growing evidence that wild animals differ in their ability to learn and retain information, these differences are partly heritable^1–3^ and they can impact fitness^4–7^. However, which cognitive abilities are beneficial and in which contexts they are beneficial has been examined in a limited number of species. Spatial memory, or the ability to memorize where objects are in space, is utilized by animals to remember where food sources are located (e.g., primates^8^, insects^9^, birds^10^, reptiles^11^). It has been hypothesized that as environments change, the ability to accurately remember where previously available food sources were in space could allow animals to more quickly return to these sources instead of enduring the cost of searching for a new patch as food availability decreases^6,8,12,13^. Therefore, environments where food availability fluctuates, such as seasonal habitats, may drive selection for more accurate spatial memory^4,14^. This hypothesized relationship between seasonally fluctuating food availability and cognition is termed the harsh environment hypothesis^4,14^.

Both latitude and elevation have been used to investigate the harsh environment hypothesis, as increases in latitude and elevation are related to more snow cover, lower temperatures, and greater seasonality, which leads to greater food scarcity and variability, as well as higher metabolic costs during winter^15–18^, but see^19^. Along these two gradients, the harsh environment hypothesis has received support in winter resident, scatter hoarding birds that store food in multiple areas and use spatial memory to return to these caches when food is scarce during the winter^20,21^. Indeed, spatial memory accuracy, and the size and neuron density of the primary brain structure responsible for spatial memory (i.e. hippocampus) increase along gradients of elevation and latitude^20,21^. Moreover, juveniles with more accurate spatial memory had greater overwinter survival, providing evidence that decreased overwinter food availability may select for more accurate spatial memory in scatter-hoarders^4^. Spatial memory could also aid non-scatter hoarders foraging in a harsh environment^6,8,12,13^ but no study, to our knowledge, has investigated whether increased environmental harshness is linked to increased spatial memory ability in non-hoarders.

Although spatial memory can contribute to foraging success, increased investment in memory also has potential costs. One hypothesized cost is proactive interference, with more accurate memories being more difficult to forget and interfering with the formation of new memories^21,22^, but see^23^. Proactive interference can lead to repeating incorrect responses instead of flexibly changing them as the situation changes or new information is presented (e.g. humans^24^, other vertebrates^22,25–27^ and invertebrates^28,29^, but see^23^). For example, an individual may repeatedly return to an empty food patch instead of learning the location of a new patch as their previous memory is interfering with their ability to learn new information. However, evidence for a correlation between increased spatial memory accuracy and increased proactive interference is not clear. Lab studies have compared rodents and birds whose neurobiology has been modified to those who were not modified (e.g. drug intake^30^, gene expression^31,32^ cannabinoid receptor blockage^33^, nutrient uptake^34^) and found concurrent changes in spatial memory and proactive interference^30–32^, but see^34^. Comparative studies have found that spatial memory accuracy is generally higher in scatter hoarders as compared to non-scatter hoarders^10,35,36^, but see^37,38^ but scatter hoarders express less, not more, proactive interference on spatial tasks than non-scatter hoarders^10,35^, but see^39^. However, two studies of mountain chickadees (*Poecile gambeli*) in the wild found that high elevation chickadees had more accurate spatial memory, but committed a greater number of errors on a previously rewarded feeder compared to low elevation birds^40,41^, In one of these studies, it was also found that the individual’s mean number of errors was positively associated with the tendency to return to the previously rewarded feeder^41^. Therefore, the evidence showing a positive correlation between spatial memory and proactive interference is not found in between-species comparisons, and only found at the phenotypic level in within-species comparisons. These phenotypic correlations may only be evidence that proactive interference increases if an individual’s spatial memory increases, and not that individuals with more accurate spatial memory, on average, have greater proactive interference compared to other individuals, otherwise known as the among-individual correlation^42,43^. There is still no direct empirical evidence from behavioural studies in wild animal populations for an among-individual correlation between spatial memory and proactive interference that would be indicative of a trade-off.

**Figure 1:**
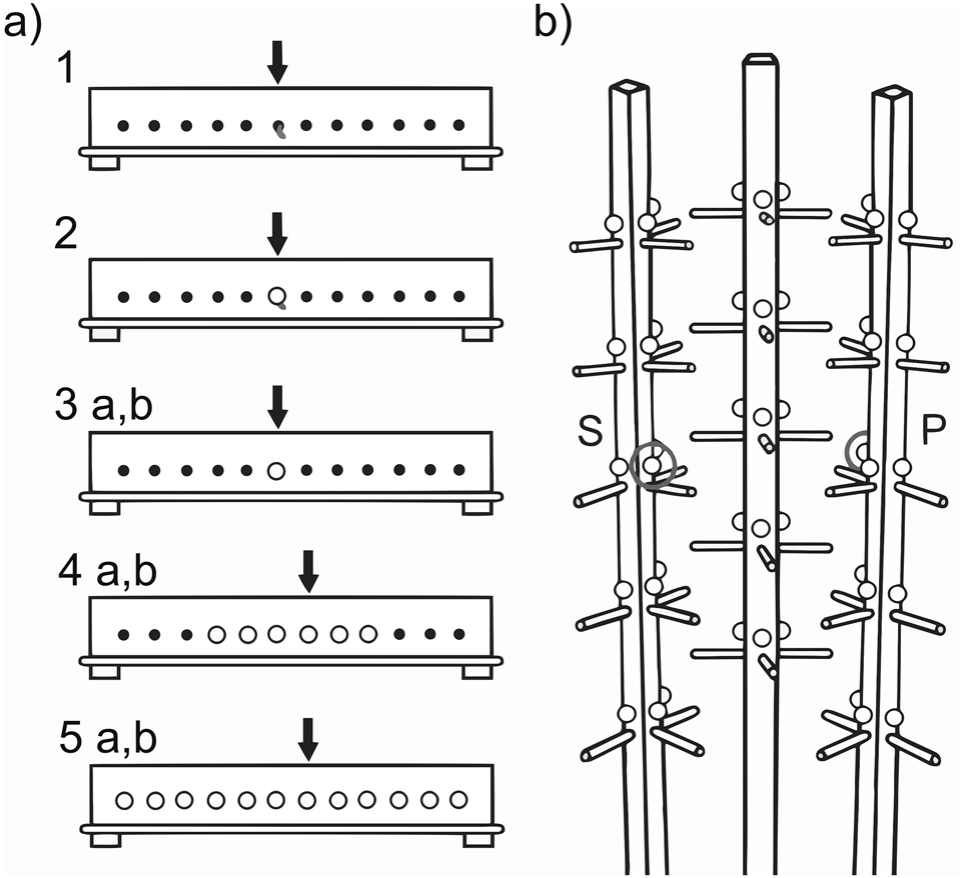
(**a**) The 5 Motor Training Stages (8 stages total) as they appeared on the training board. The black dots represent empty holes. The white dots represent holes covered by white, acrylic 0.5 inch pompoms. The arrows denote the location of the mealworm (*Tenebrio molitor*) reward. The mealworm reward is pictured uncovered in Stage 1, half covered in Stage 2, and completely covered by a pompom after Stage 3. In each of the stages, the bird had to consume the mealworm reward from the training panel to progress to the next stage. If it failed the stage (i.e., did not consume the meal worm within 15 min), it regressed to the previous stage. (**b**) Location of the mealworm reward during the spatial memory trials (S) and proactive interference trials (P). The location of the worm alternated sides across cages, and only one side is pictured here. It proceeded with the worm having no pompom over it (information stage 1). If the bird successfully retrieved the worm, another worm was placed in the hole (information stage 2–1) and two mealworms were placed in the hole if the initial worm was not retrieved (information stage 2–2). The bird continued onto information stage 3 if the mealworms were consumed or stayed at information stage 2–2 if they were not. For information stage 3, the bird retrieved a mealworm half covered by a pompom, then retrieved a mealworm completely covered by a single pompom for information stages 4–5. Every hole was covered with a pompom during the spatial memory and proactive interference trials.

In this study, we compared the performance of non-scatter hoarding, great tits (*Parus major*), that feed on patches of seeds during the winter^44^, from several high and low elevation sites that differ in harshness^16,45,46^ on a spatial memory task and a single spatial reversal task designed to measure proactive interference^21^ (Fig. 1b). The spatial memory portion of this task consists of an information stages where birds are shown the location of a food reward in one tree out of three^47^. Their memory for the location of this reward is then tested over 7 trials with memory accuracy being counted as the number of errors before finding the food reward^47,48^. The reversal task to measure proactive interference consists of a single information stage wherein the food reward was moved to a previously unrewarded tree, and 5 trials to measure the ratio of errors made on the previously rewarded tree over the other two trees^41^. We predict that high elevation birds will commit fewer errors than low elevation birds on a spatial memory task if spatial memory aids in foraging in a harsh environment. We predict that if there is a relationship between accurate memory and increased proactive interference, during the reversal, high elevation birds will also commit a greater ratio of errors on the previously rewarded tree than low elevation birds (i.e., greater proactive interference). Finally, if accurate memory correlates with greater proactive interference, we predict that performance on both tasks will negatively co-vary at the among-individual level^42^. In order to assess this correlation, we also quantify repeatability (i.e., consistency of individual differences) of accuracy during the spatial memory and proactive interference trials^49^.

## Results

### Motor training

All high and low elevation birds approached the task and consumed a half-covered mealworm (Stage 2; Fig. 1a). There was no significant difference between elevations in the number of attempts to pass consume a half-covered mealworm (Mean ± s.d.: High: 1.32 ± 1.72; Low: 1.33 ± 0.79; Wilcoxon rank sum test, high vs. low: W = 531, P = 0.104, n = 70). There was no significant difference between elevations in the number of stages required to successfully pass motor training (Mean ± s.d.: High: 11.3 ± 3.68; Low: 11.2 ± 3.21; Wilcoxon rank sum test, high vs. low: W = 537, P = 0.759, n = 67). There was no significant difference between elevations in the proportion of birds that passed motor training (High: 33/34; Low: 34/36; Fisher’s exact test, Contingency table: CI = [0.008, 10.434], odds ratio = 0.520, P = 1). Therefore, high and low elevation birds were both successfully trained to remove pompoms covering food rewards and exhibited no apparent difference in motivation to consume the mealworms.

### Spatial memory

**Figure 2:**
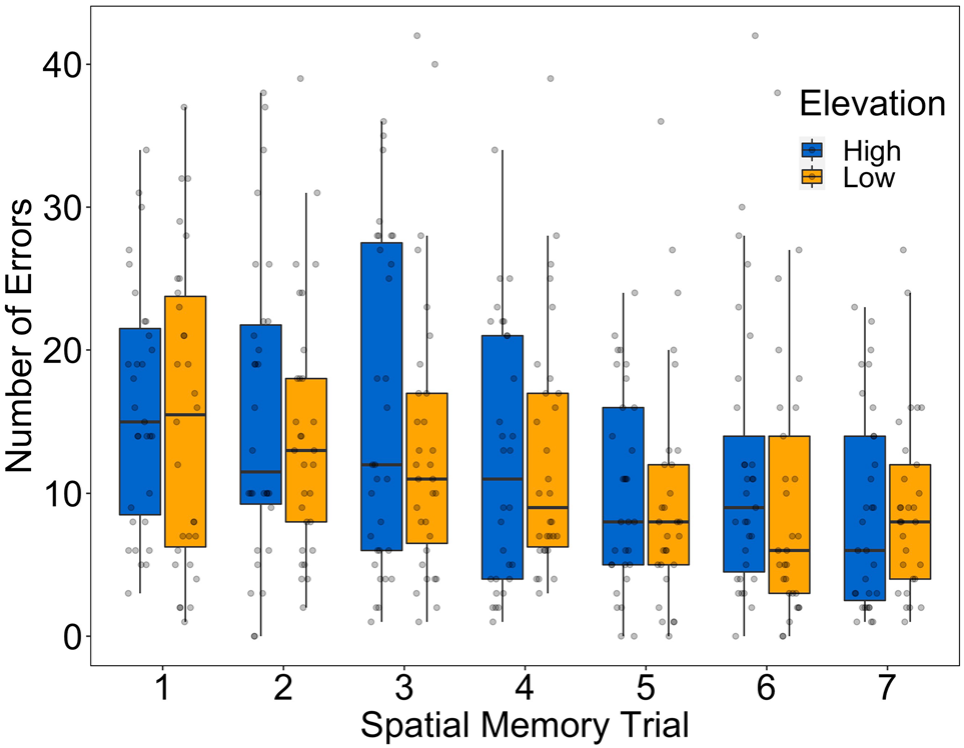
Boxplot of the untransformed number of spatial memory errors across spatial memory trials. High elevation birds are in blue, low elevation birds are in orange. Raw number of errors are plotted in grey.

The birds made significantly fewer errors than expected by random searching (chance = 23 following negative hypergeometric distribution; One-tailed Wilcoxon signed-rank test, N = 62, mean ± s.d. = 12.478 ± 5.397, P < 0.001). This indicates the birds had learned the location of the reward. Trial number was significant and negative, indicating that birds improved in accuracy over trials. Elevation was non-significant (Table 1; Fig. 2). This indicates that high and low elevation birds did not differ in their spatial memory accuracy.

**Table 1:** Predictors of the log transformed number of errors made by birds (n = 62; n = 423 trials) across 7 spatial memory trials fitted with a linear mixed effect model with trial, capture order, intertrial interval (minutes), elevation (high/low), sex (male/female), age (juvenile/adult), rewarded side of the tree (left/right), and observer (EH, JH, AR) included as fixed effects.

Predictors	Estimate ± SE	F-statistic	*P*
Intercept	2.162 ± 0.258		
Trial	− 0.207 ± 0.033	38.947	< .0001
Elevation (low)	− 0.137 ± 0.126	1.177	0.283
Sex (male)	0.022 ± 0.124	0.032	0.859
Age (juvenile)	− 0.048 ± 0.138	0.121	0.729
Capture order	0.235 ± 0.113	4.334	0.042
Intertrial interval	0.020 ± 0.033	0.366	0.546
Correct side (right)	− 0.114 ± 0.122	0.868	0.356
Observer (EH)	0.446 ± 0.270	1.370	0.263
Observer (JH)	0.242 ± 0.255		

Bird ID was included as a random intercept.

### Proactive interference

**Figure 3:**
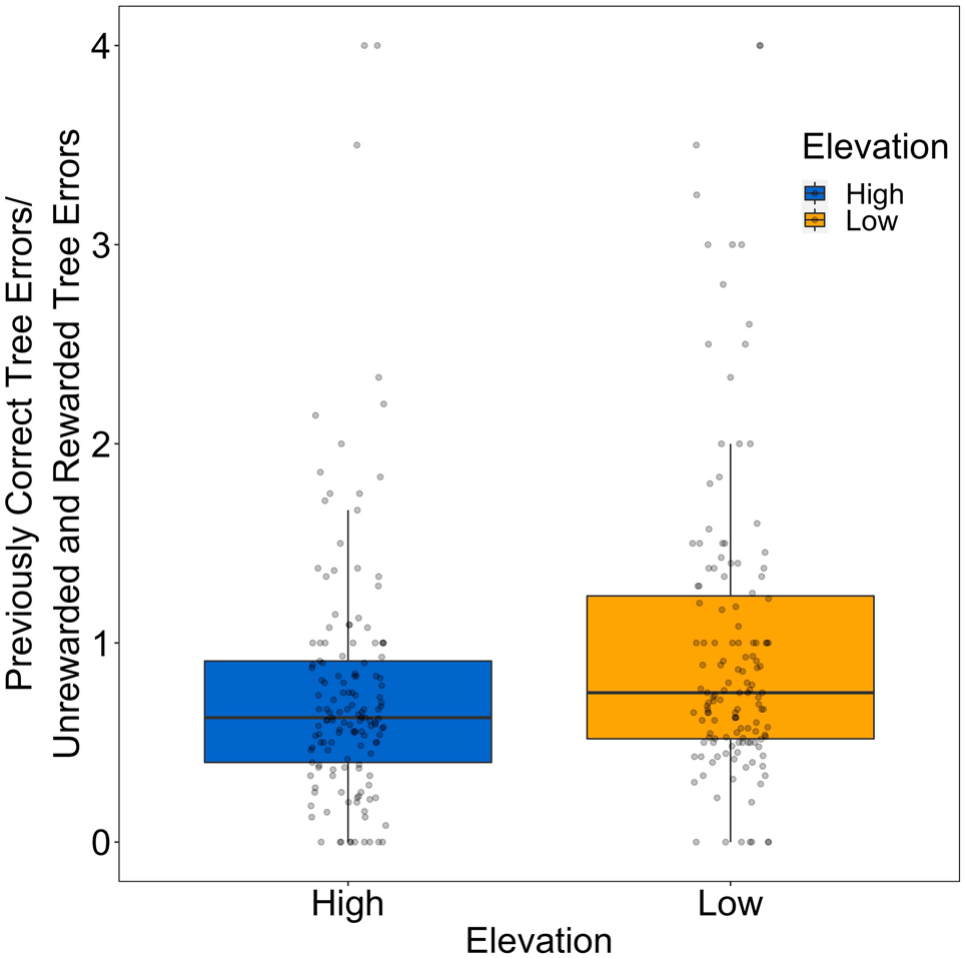
Boxplot of the untransformed previously correct tree errors/unrewarded and rewarded tree errors for high (blue) and low (orange) elevation birds. Raw number of errors are plotted in grey.

The mean ratio of errors to the previously rewarded tree (previously rewarded tree/rewarded and unrewarded tree) was significantly higher than expected by random sampling (chance = 0.5; One-tailed Wilcoxon signed-rank test, N = 59, mean ± s.d. = 0.879 ± 0.448, P < 0.001). The number of errors was significantly higher in the first proactive interference trial than in the first spatial memory trial (One-tailed paired t-test, t = − 4.799, CI = − 6.309, mean of the difference = − 9.684, P < 0.001; N = 57), suggesting that the birds did show evidence for proactive interference. All fixed effects, including elevation, were non-significant (Table 2; Fig. 3). This indicates that high and low elevation birds did not differ in their intensity of proactive interference.

**Table 2:** Predictors of the log transformed ratio of errors made by birds (n = 59; n = 284 trials) across 5 proactive interference trials fitted with a linear mixed effect model with trial, capture order, intertrial interval (minutes), and elevation (high/low), sex (male/female), age (juvenile/adult) rewarded side of the tree (left/right), and observer (EH, JH, AR) included as fixed effects.

Predictors	Estimate ± SE	F-statistic	*P*
Intercept	0.610 ± 0.084		
Trial	0.013 ± 0.017	0.579	0.448
Elevation (low)	0.098 ± 0.059	2.790	0.101
Sex (male)	− 0.013 ± 0.059	0.046	0.831
Age (juvenile)	− 0.069 ± 0.066	1.117	0.295
Capture order	0.024 ± 0.029	0.681	0.413
Intertrial interval	0.007 ± 0.017	0.182	0.670
Correct side (right)	− 0.083 ± 0.058	2.059	0.157
Observer (EH)	− 0.096 ± 0.147	0.307	0.736
Observer (JH)	0.029 ± 0.076		

Bird ID was included as a random intercept.

### Among-individual trade-off

**Figure 4:**
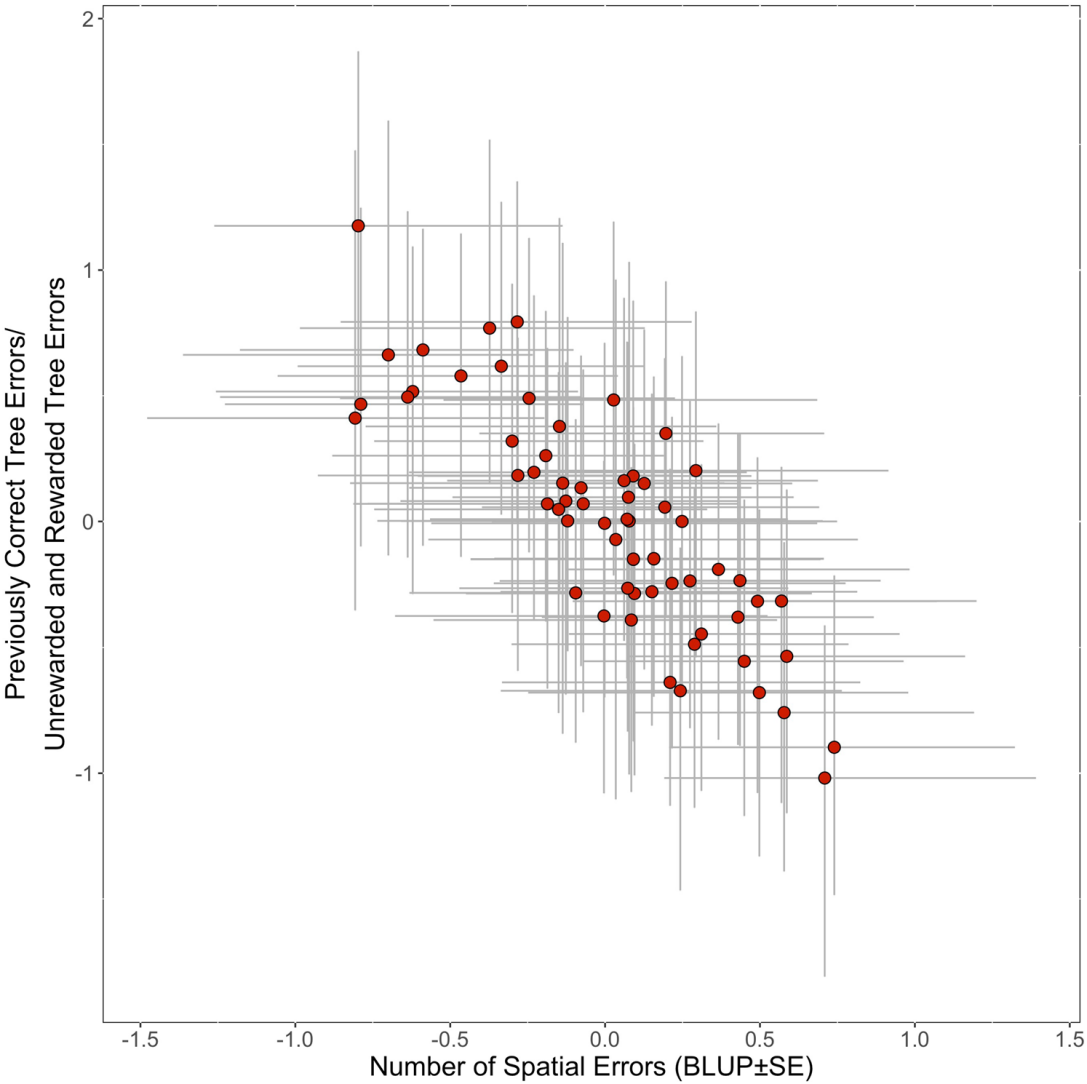
Among-individual correlation (red) between the standardized number of spatial errors and the standardized number of errors made to the previously rewarded tree/unrewarded and rewarded trees in great tits (*Parus major*) from high and low elevations. Individual deviations from the population mean are illustrated using best linear unbiased predictors (BLUP ± SE) associated with the random effect of bird identity.

The number of errors was significantly and moderately repeatable across spatial memory trials (R = 0.232 ± 0.053, CI = [0.135, 0.342], P < 0.001), as well as across proactive interference trials (R = 0.295 ± 0.068, CI = [0.170, 0.426], P < 0.001). There was strong evidence that the number of errors in the spatial memory task was negatively correlated to the ratio of errors in the proactive interference task at the among-individual level (r_ind_ = − 0.677 ± 0.145; 95% CI = [− 0933, − 0.387]; Fig. 4). This indicates that individuals who performed more accurately on the spatial memory task showed greater proactive interference.

## Discussion

Although we know that there are individual differences in wild animal cognition, and these differences may affect fitness^4–7^, we still do not fully understand how the environment may impact cognition in natural populations^13,50,51^. Harsh environments may be one driver of individual differences in cognition^14,15^. Accurate spatial memory could aid foraging in harsh environments, but it is predicted to inhibit the formation of new memories, creating a trade-off^21^. We explored these relationships for the first time in a wild population of a non-hoarding species. We found no differences in spatial memory accuracy or proactive interference performance among great tits from low and high elevations which differ in harshness, but we did find that birds that were more accurate on a spatial task also had greater proactive interference. This among-individual correlation between performance in both tasks reveals a trade-off at the level at which selection can act.

Studies of the harsh environment hypothesis have consistently found that spatial memory is more accurate in scatter hoarders from high than low elevations^21^, and we predicted the same relationship in non-scatter hoarders. However, we did not find a significant difference in spatial memory accuracy between high and low elevation great tits. A first possible explanation is that the two elevations we sampled do not differ enough in harshness. However, this is unlikely as previous studies that sampled birds from the same population and elevations found a significant cognitive difference (i.e., laboratory test of serial reversal learning^16^), as well as differences in breeding phenology^52^ and parental care^45^ between high and low elevation birds. Therefore, some environmental difference between high and low elevations seems to be driving behavioural differences. Second, the differences between our results and those from previous studies could be due to a difference in selection pressure between scatter hoarders and non-scatter hoarders. Scatter hoarders, specifically mountain and black-capped chickadees, from harsher environments have a higher propensity to cache food than scatter hoarders from less harsh habitats^14,53^. Caching utilizes spatial memory and the greater need to remember caches creates strong selection for spatial memory as harshness increases^4,5,14,54,55^. Non-scatter hoarders may still utilize accurate spatial memory when remembering and returning to food patches^8–11^ but this need may be similar to that of scatter hoarders that need to find and return to the food when initially foraging for food to cache. Therefore, the adaptive value of accurate spatial memory may not be as high for non-scatter hoarders and any differences among elevations may be small. A third potential explanation is that greater spatial memory accuracy may also be just as helpful in low than high harshness environments. For example, our lower elevation field sites are characterized as having more diversity in food sources than high elevation^45^. In areas of high diversity, it may be beneficial to remember where the high quality food items are and return to the best food source^8^. Finally, it is possible that our spatial memory task was not difficult enough to detect a difference between elevations. For instance, we used retention intervals of 1 h as well as overnight. Increasing the retention interval to weeks instead of hours or days may make the task difficult enough for even small differences in spatial memory across elevations to show (e.g., 17 days^56^). Testing for a correlation between spatial memory performance and over-winter survival may allow for a better understanding of spatial memory’s usefulness to non-scatter hoarders^5^.

We predicted that high elevation great tits should suffer greater proactive interference and make a larger proportion of errors on the previously correct tree compared with low elevation birds. Instead, and in accordance with a lack of spatial memory accuracy differences, we found no difference between high and low elevation birds in their proactive interference. Interestingly, in a previous study on great tits collected from the same population, we found that birds from high elevation performed less accurately on a serial spatial reversal learning task than low elevation great tits^16^. We surmised that one potential explanation for this result was that spatial memory may have been more accurate in high elevation great tits, which would lead to greater spatial proactive interference and worse reversal performance, as found in the scatter hoarding system^21,41^. Given that there is no difference in proactive interference between high and low elevation birds in the current study, we have preliminary evidence to rule out this explanation. Instead, our previous results may have been due to differences in win-stay/lose-shift rule learning between elevations, where the animal is not memorizing associations per se but is changing its choice when it is incorrect and maintaining that choice when it is correct^57^. As the great tits did not reach a single error switch in our serial reversal learning task, we were unable to measure this^16^. This explanation remains to be tested empirically.

We found a positive among-individual correlation between spatial memory accuracy and proactive interference. This positive among-individual correlation indicates that individuals that have more accurate spatial memory on average, also have higher proactive interference on average^43^. This correlation would traditionally be assessed at the unpartitioned, phenotypic level, by collecting one measure of spatial memory, and one measure of proactive interference for multiple individuals. However, phenotypic correlations are influenced by within-individual variance, which reflects how two traits change with each other within the individual over repeated measurements. For example, if an individual great tit’s spatial memory accuracy increases with state or age, a positive within-individual correlation would indicate that its proactive interference should also increase with state or age^42,43^. To avoid this ‘individual gambit’, multiple measures of each test are used to partition variance to among-individual and within-individual levels, and directly assess among-individual correlation^43^. We found a positive among-individual correlation between spatial memory accuracy and proactive interference performance with great tits who, on average, made a lower number of errors during their spatial memory trials, also on average made a greater proportion of their errors on the tree that was previously rewarded during the spatial task. In other words, birds who remember well also have a difficult time forgetting and learning a new reward location. To our knowledge, this is the first examination of an among-individual correlation between spatial memory accuracy and proactive interference in wild animals.

Our among-individual correlation generally agrees with other lab experiments^30–32^ and field studies^40,41^, that show evidence for a trade-off between spatial memory accuracy and proactive interference performance. However, our population comparisons did not match the results found in the scatter hoarding systems^40,41^. We believe this indicates that although there are no differences between high and low elevation great tits in either of these behaviours, there is preliminary evidence that this trade-off is present in the overall population. If a change in selection pressure occurs that leads to an increase in spatial memory accuracy in either high or low elevation great tits, their proactive interference could be expected to change in kind. However, we do caution that this result would be more robust with additional testing to see if this relationship holds. Our multiple measures for spatial memory and proactive interference came from the same cognitive task and may thus suffer from a lack of independence. In the future, a more robust test should alternate measuring spatial memory and proactive interference, ideally using a different experimental set-up the second time (i.e., contextual repeatability^58^ e.g., spatial task and reversal using a set of automated feeders^59^). Finally, increasing the number of cues tested (e.g., spatial and colour) could increase our understanding of the relationship between learning and proactive interference in general.

Overall, we did not find any population differences in either spatial memory accuracy or proactive interference performance measures. We found that individual great tits’ proactive interference and spatial memory accuracy are both significantly and moderately repeatable and are traded-off at the among-individual level. Therefore, our results show that spatial memory may not be under increased selection at high elevations as it seems to be in some scatter-hoarding birds, but the material is there for selection to concurrently act upon spatial memory and proactive interference. Selection may act differently depending on a species’ or population’s functional behaviour and ecology, and cognitive ecology research should continue to open up the breadth of study systems examined.

## Methods

### Capture and housing

Wild great tits (*Parus major*) were captured from 3 high (800–900 m; n = 34; n = 17 males, n = 17 females, n = 22 juveniles, n = 12 adults) and 3 low elevation sites (400–500 m; n = 36; n = 22 males, n = 14 females, n = 24 juveniles, n = 12 adults) near the Experimental and Theoretical Ecology Research Station in Moulis, France between October 24, 2017, and February 25, 2018 (See Supplementary Fig. S1). High elevation sites are characterized by longer weekly snow cover, and lower temperatures relative to low elevation sites^16,45,46,52^. Great tits were captured in batches of 4–6 individuals, using mist nests and marked with a CRBPO (Centre de Recherches sur la Biologie des Populations d’Oiseaux) metal band. We used plumage to sex (male/female) and age (juvenile/adult) great tits^60^. Birds that experienced a previous cognitive test were released and not used in testing. Birds were transported to outdoor aviaries in cloth bags and housed individually (1 × 4 × 3 m) in every second cage to visually isolate them. High and low elevation birds were housed in the same cages, on the same side of the aviary, but the experimenters were not blind to the bird’s identity during placement. Each aviary contained foliage for cover in the non-testing area, 2 roosting boxes, and 2 horizontal perches between the foliage and testing area.

### Acclimatization

For 6 days after capture, birds were acclimated to the aviary and testing environment (See Supplementary Table S4). The birds had access to ad libitum black oil sunflower seeds, fat balls, meal worms, and water. A small heated (25 °C) room inside of the aviary building was left open and contained a second source of ad libitum food and water and constant light for the first two days to encourage feeding. Three un-baited testing trees and one un-baited motor training panel were in the aviary to acclimatize the birds to the testing devices. The testing trees, motor training board, and food were removed during the final night of acclimatization (day 6). To reduce stress to the birds caused by recapture, weight was not measured during acclimatization or during testing. All training and testing occurred concurrently for each batch of birds.

### Motor training

On day 7, the birds were trained to approach and remove a pompom (0.5 inch diameter white acrylic ball) from a 0.5 cm hole and retrieve a mealworm reward hidden underneath the pompom across 8 stages on a training board (Fig. 1a; See Supplementary Table S4). 15-min motor training sessions occurred one after another from 08 h to 11 h 30. Ad libitum food was given from 11 h 30 to 12 h 30. Training resumed until 16 h 00, or until the birds passed motor training. Ad libitum food was returned to the cage and the motor training panel was removed afterwards. Fall and Winter birds underwent a slightly different motor training protocol due to slight differences in methodology. When fall birds failed to pass stage 2, they did not revert to stage 1 but rather stayed at stage 2 (low: n = 3, high: n = 1). Also, one bird experienced 5a twice after 3b, was returned to 4a and then proceeded to pass 4a–5b. We included these extra trials in the motor training analysis. Removing this individual (bird id: 123) did not qualitatively change the results. All birds that successfully passed motor training were kept in the analysis and began information traits on day 8 (see Supplementary Fig. S2, Table S3).

### Information trial, spatial memory, and proactive interference

The night of day 7, the 3 testing trees were added back to the cage, and the food was removed (Fig. 1b; see Supplementary Table S4). On day 8 (07h00), birds had to complete 5 information trials before proceeding to the spatial memory task. During the information trials, the birds learned the rewarded location by repeatedly retrieving a worm from the same location on one of the testing trees: twice the worm was uncovered, once half covered, and twice completely covered by a pompom. All birds that consumed all the worms during the information trials were kept in the analysis and proceeded to spatial memory tests (see Supplementary Fig. S2, Table S3).

The spatial memory task^47,48^ started on day 8 at 10 h (Fig. 1b; See Supplementary Table S4). Before each spatial memory trial, birds were food deprived for 30-min. To decrease the usefulness of potential social cues from the experimenters, we mimed placing the worm into each hole by covering the hole with a hand and motioning as if placing the worm in the hole underneath before placing the pompom. Pompoms were placed into all of the trees’ holes (n = 45) in the same order throughout trials, and a mealworm was placed into the same rewarded hole as in the information stage (Fig. 1b). Birds had 1 h to find the worm and all pompoms pulled before finding the worm were considered errors^14,47^. Ad libitum food was returned after the first spatial memory trial for 30-min. Two more spatial memory trials followed, with a 30-min deprivation period occurring in between. At the end of these two trials, ad libitum food was returned to the cages, and removed at night.

Four spatial memory trials occurred on day 9 (see Supplementary Table S4). The fourth trial started at 08 h 00, and trial 5 followed after a 30-min deprivation. The rest of the trials followed the same schedule as the previous day. If the worm was not found during a trial, it was left in the rewarded hole after the pompoms were removed. Birds that did not consume the open mealworm were excluded from further trials (see Supplementary Fig. S2, Table S3). If the worm was found, the intertrial interval was calculated from when the worm was found, and only the trial where the bird did not find the worm was excluded. If a bird failed to consume the worm twice during the spatial memory tests, we only kept trials up to the second missed trial as we assumed the bird was not motivated to complete the task.

Proactive interference trials followed the same protocol as the second day of the spatial memory task (day 10; see Supplementary Table S4, Video S9). However, the reward was now located on the tree opposite to the previously rewarded tree, in an inward facing hole (Fig. 1b). The first trial was a single information stage followed by 3 proactive interference trials. Two more proactive interference trials followed on the next day (day 11). Errors made on the previously rewarded tree indicate that birds did not extinguish the positive association with the previously rewarded location, while errors on the other two trees are assumed to be due to the newly learned association with the currently rewarded tree, or exploration errors made to the never rewarded tree. Therefore, proactive interference was quantified as the ratio of errors made on the previously rewarded tree, over the errors made on the other 2 trees^41^. Some birds became acclimated to the tester and would start removing pompoms before all pompoms were placed in the trees (n = 4 trials; Supplementary Table S3). We did not count these errors in our analysis, but results were qualitatively the same with or without these trials.

### Video analysis

Data from the information trial, spatial memory and proactive interference trials were extracted using BORIS video analysis software by 3 observers (EH, AR, JH)^61^. A blind procedure was used with observers watching muted videos labelled by dates or batch with no identifying information viewable on the screen. Intertrial interval was quantified as the time difference (minutes) between the moment when a bird found a mealworm in the previous trial, and when the experimenter left the cage after preparing it for the start of the next trial. Videos for some birds were lost (n = 2 birds); only their trials up to the missing videos were kept in the analysis. (see Supplementary Figs. S2, S3). The number of errors for some trials exceeded the possible number of errors (> 15 errors on a tree: n = 9/284 PI trials, > 45 total errors: n = 1/284 PI trials). These extra errors were kept in the analysis as we assume the observer randomly overcounted errors across all high and low elevation birds, and only removing detectable overcounts would be artificially lowering only high error count videos.

### Statistical analysis

We compared motor training speed between elevations by comparing the number of trials it took to consume a worm half covered by a pompom (Stage 2; Fig. 1a), as well as the number of trials to pass motor training using a non-paired Wilcoxon Rank Sum Test as the data distribution did not fit the assumption of normality. We also compared the proportion of high and low elevation birds that passed motor training using a Fisher’s exact test.

We analyzed whether the birds had learned the location of the reward during the spatial memory trials by comparing the mean number of spatial errors to the mean number of errors predicted by random searching using a Wilcoxon signed rank test^62^. We analyzed the relationship between elevation and spatial memory using a linear mixed model (LMM; lme4 1.1–25^63^, lmerTest3.1–3^64^ R Version 4.0.3^65^) with log transformed number of errors as the response to meet the assumption of normality of the residuals^66^. Elevation (high/low), age (juvenile/adult), sex (male/female), capture order (1–12), which tree the reward was on (left/right), trial number (1–7), video observer (EH, AR, JH) and inter-trial interval (minutes) were included as fixed effects. Bird ID was included as a random intercept. To control for the effect of capture site we included site as a random effect, but the model would not converge. Therefore, we ran a separate high and low elevation models and included site as a fixed effect. Site was not significant in either model and it was excluded from further analysis (see Supplementary Tables S5, S6).

We analyzed whether the birds experienced proactive interference by comparing the mean ratio of errors to the ratio of errors that we would expect given random sampling of the three trees [(1/3)/(2/3)] using a Wilcoxon signed rank test. We also compared trial 1 errors between the spatial memory task and proactive interference task using a one-tailed, paired sample t-test. We analyzed the relationship between elevation and proactive interference using an LMM with the log transformed ratio of errors to meet the assumption of normality of the residuals. We utilized the same fixed and random effects as the above model except trial number went from 1 to 5. The fit was singular with site as a random effect. Therefore, we ran the separate high and low elevation models and included site as a fixed effect. The high elevation model would not run with observer included and so it was dropped from the model. Site was not significant in either model and was excluded from further analysis (see Supplementary Tables S7, S8). All continuous predictor variables were standardized by grand mean centering and dividing by 1 standard deviation. Assumptions of normality and homogeneity were visually assessed using histograms, Q-Q plots, and residual versus fitted plots, respectively. The analysis was not performed blind and sample sizes were not calculated a priori.

Adjusted repeatabilities^67^ were calculated using the same models as the LMMs without sex and age using rptR (rptR 0.9.22^68^). A multivariate mixed model was utilized to calculate the among-individual covariance between performance on the spatial memory and proactive interference measures (MCMCglmm 2.29^69^). The log transformed number of errors from the spatial memory trials and the log transformed ratio of errors from the proactive interference trials were included as traits with Gaussian error structures. We included the same fixed effects as above. Bird ID was included as a random effect for both traits. Family was defined as ‘Gaussian’ and residual variance at the limit was set to 1. The random effect variance structure (*G*) used in the prior included a variance set to 1 and a degree of belief (nu) set to 0.002. Burn in was set to 20,000, the number of iterations was 420,000, thin was set to 100^70^. Convergence of the model was assessed by visual inspection of traces.

### Ethics

Trapping and marking of wild great tits was performed under permits from the French ringing office (CRBPO, project 576; permit 13619). Capture and holding birds from the wild was approved by the Région Midi-Pyrenées (DIREN, n°2012-07) in the Moulis experimental aviaries (Préfecture de l’Ariège, institutional permit n°SA-12-MC-054; Préfecture de l’Ariège, Certificat de Capacite, n°09-321). This study was approved by the Animal Care Committee at the University of Ottawa (protocol: 1758). Testing complied with the ARRIVE Essential 10 guidelines^71^. All methods were performed in accordance with the relevant guidelines and regulations.

## Supplementary Information

Supplementary Information 1.

Supplementary Information 2.

Supplementary Video 1.

## Data Availability

All data generated or analysed during this study, and the code used to analyze the data, are included in this published article and its Supplementary Information (Supplementary Info File, Supplementary Code and Dataset File, Supplementary Video S9).

## References

[1] Croston R, Branch CL, Kozlovsky DY, Dukas R, Pravosudov VV (2015). The importance of heritability estimates for understanding the evolution of cognition: A response to comments on Croston et al. Behav. Ecol..

[2] Langley EJG (2020). Heritability and correlations among learning and inhibitory control traits. Behav. Ecol..

[3] Boogert NJ, Madden JR, Morand-Ferron J, Thornton A (2018). Measuring and understanding individual differences in cognition. Philos. Trans. R. Soc. B..

[4] Sonnenberg BR, Branch CL, Pitera AM, Bridge E, Pravosudov VV (2019). Natural selection and spatial cognition in wild food-caching mountain chickadees. Curr. Biol..

[5] Benedict LM (2020). Elevation-related differences in annual survival of adult food-caching mountain chickadees are consistent with natural selection on spatial cognition. Behav. Ecol. Sociobiol..

[6] Shaw RC, MacKinlay RD, Clayton NS, Burns KC (2019). Memory performance influences male reproductive success in a wild bird. Curr. Biol..

[7] Cauchoix M, Chaine AS (2016). How can we study the evolution of animal minds?. Front. Psychol..

[8] Janmaat KRL (2016). Spatio-temporal complexity of chimpanzee food: How cognitive adaptations can counteract the ephemeral nature of ripe fruit. Am. J. Primatol..

[9] Collett M, Chittka L, Collett TS (2013). Spatial memory in insect navigation. Curr. Biol..

[10] Hampton RR, Shettleworth SJ (1996). Hippocampus and memory in a food-storing and in a nonstoring bird species. Behav. Neurosci..

[11] LaDage LD, Roth TC, Cerjanic AM, Sinervo B, Pravosudov VV (2012). Spatial memory: Are lizards really deficient?. Biol. Lett..

[12] Milton K (1981). Distribution patterns of tropical plant foods as an evolutionary stimulus to primate mental development. Am. Anthropol..

[13] Thornton A, Boogert NJ (2019). Animal cognition: The benefits of remembering. Curr. Biol..

[14] Pravosudov VV, Clayton NS (2002). A test of the adaptive specialization hypothesis: Population differences in caching, memory, and the hippocampus in black-capped chickadees (*Poecile atricapilla*). Behav. Neurosci..

[15] Morand-Ferron J, Hermer E, Jones TB, Thompson MJ (2019). Environmental variability, the value of information, and learning in winter residents. Anim. Behav..

[16] Hermer E, Cauchoix M, Chaine AS, Morand-Ferron J (2018). Elevation-related difference in serial reversal learning ability in a nonscatter hoarding passerine. Behav. Ecol..

[17] Boyle AW, Sandercock BK, Martin K (2016). Patterns and drivers of intraspecific variation in avian life history along elevational gradients: A meta-analysis. Biol. Rev..

[18] Roth TC, Pravosudov VV (2009). Hippocampal volumes and neuron numbers increase along a gradient of environmental harshness: A large-scale comparison. Proc. R. Soc. B.

[19] Körner C (2007). The use of ‘altitude’ in ecological research. Trends Ecol. Evol..

[20] Roth TC, LaDage LD, Pravosudov VV (2010). Learning capabilities enhanced in harsh environments: A common garden approach. Proc. R. Soc. B.

[21] Tello-Ramos MC, Branch CL, Kozlovsky DY, Pitera AM, Pravosudov VV (2018). Spatial memory and cognitive flexibility trade-offs: to be or not to be flexible, that is the question. Anim. Behav..

[22] Gonzalez RC, Behrend ER, Bitterman ME (1967). Reversal learning and forgetting in bird and fish. Science.

[23] Strang CG, Sherry DF (2014). Serial reversal learning in bumblebees (*Bombus impatiens*). Anim. Cogn..

[24] Herszage J, Censor N (2018). Modulation of learning and memory: A shared framework for interference and generalization. Neuroscience.

[25] Squier LH (1969). Reversal learning improvement in the fish *Astronotus ocellatus* (Oscar). Psychon. Sci..

[26] Miyashita Y, Nakajima S, Imada H (2000). Differential outcome effect in the horse. J. Exp. Anal. Behav..

[27] Missaire M (2017). Long-term effects of interference on short-term memory performance in the rat. PLoS ONE.

[28] Bublitz A, Weinhold SR, Strobel S, Dehnhardt G, Hanke FD (2017). Reconsideration of serial visual reversal learning in octopus (*Octopus vulgaris*) from a methodological perspective. Front. Physiol..

[29] Chittka L (1998). Sensorimotor learning in bumblebees: Long-term retention and reversal training. J. Exp. Biol..

[30] Chrobak JJ, Hinman JR, Sabolek HR (2008). Revealing past memories: Proactive interference and ketamine-induced memory deficits. J. Neurosci..

[31] Malleret G (2010). Bidirectional regulation of hippocampal long-term synaptic plasticity and its influence on opposing forms of memory. J. Neurosci..

[32] Joseph MA (2015). Differential involvement of the dentate gyrus in adaptive forgetting in the rat. PLoS ONE.

[33] Shiflett MW, Rankin AZ, Tomaszycki ML, DeVoogd TJ (2004). Cannabinoid inhibition improves memory in food-storing birds, but with a cost. Proc. R. Soc. B..

[34] Meck WH, Williams CL (1999). Choline supplementation during prenatal development reduces proactive interference in spatial memory. Dev. Brain Res..

[35] Clayton NS, Krebs JR (1994). One-trial associative memory: Comparison of food-storing and nonstoring species of birds. Anim. Learn. Behav..

[36] McGregor A, Healy SD (1999). Spatial accuracy in food-storing and nonstoring birds. Anim. Behav..

[37] Healy SD (1995). Memory for objects and positions: Delayed non-matching-to-sample in storing and non-storing tits. Q. J. Exp. Psychol. Sect. B.

[38] Healy SD, Krebs JR (1992). Delayed-matching-to-sample by marsh tits and great tits. Q. J. Exp. Psychol. B.

[39] Hampton RR, Shettleworth SJ, Westwood RP (1998). Proactive interference, recency, and associative strength: Comparisons of black-capped chickadees and dark-eyed juncos. Anim. Learn. Behav..

[40] Tello-Ramos MC (2018). Memory in wild mountain chickadees from different elevations: Comparing first-year birds with older survivors. Anim. Behav..

[41] Croston R (2017). Predictably harsh environment is associated with reduced cognitive flexibility in wild food-caching mountain chickadees. Anim. Behav..

[42] Careau V, Wilson RS (2017). Of uberfleas and krakens: Detecting trade-offs using mixed models. Integr. Comp. Biol..

[43] Niemelä PT, Dingemanse NJ (2018). On the usage of single measurements in behavioural ecology research on individual differences. Anim. Behav..

[44] Gosler AG (1993). The Great Tit.

[45] Lejeune L (2019). Environmental effects on parental care visitation patterns in blue tits *Cyanistes caeruleus*. Front. Ecol. Evol..

[46] Bründl AC (2019). Experimentally induced increases in fecundity lead to greater nestling care in blue tits. Proc. R. Soc. B..

[47] Thompson MJ, Morand-Ferron J (2019). Food caching in city birds: Urbanization and exploration do not predict spatial memory in scatter hoarders. Anim. Cogn..

[48] Roth TC, LaDage LD, Freas CA, Pravosudov VV (2012). Variation in memory and the hippocampus across populations from different climates: A common garden approach. Proc. R. Soc. B.

[49] Griffin AS, Guillette LM, Healy SD (2015). Cognition and personality: An analysis of an emerging field. Trends Ecol. Evol..

[50] Ashton BJ, Thornton A, Ridley AR (2018). An intraspecific appraisal of the social intelligence hypothesis. Philos. Trans. R. Soc. B..

[51] Croston R, Branch CL, Kozlovsky DY, Dukas R, Pravosudov VV (2015). Heritability and the evolution of cognitive traits. Behav. Ecol..

[52] Bründl AC (2020). Elevational gradients as a model for understanding associations among temperature, breeding phenology and success. Front. Ecol. Evol..

[53] Freas CA, LaDage LD, Roth TC, Pravosudov VV (2012). Elevation-related differences in memory and the hippocampus in mountain chickadees, *Poecile gambeli*. Anim. Behav..

[54] Pravosudov VV, Roth TC (2013). Cognitive ecology of food hoarding: The evolution of spatial memory and the hippocampus. Annu. Rev. Ecol. Evol. Syst..

[55] Croston R (2015). Potential mechanisms driving population variation in spatial memory and the hippocampus in food-caching chickadees. Integr. Comp. Biol..

[56] Kozlovsky DY, Weissgerber EA, Pravosudov VV (2017). What makes specialized food-caching mountain chickadees successful city slickers?. Proc. R. Soc. B.

[57] Izquierdo A, Brigman JL, Radke AK, Rudebeck PH, Holmes A (2017). The neural basis of reversal learning: An updated perspective. Neuroscience.

[58] Cauchoix M (2018). The repeatability of cognitive performance: A meta-analysis. Neuroscience.

[59] Croston R (2016). Individual variation in spatial memory performance in wild mountain chickadees from different elevations. Anim. Behav..

[60] Svensson L (1992). Identification Guide to European Passerines.

[61] Friard O, Gamba M (2016). BORIS: A free, versatile open-source event-logging software for video/audio coding and live observations. Methods Ecol. Evol..

[62] Tillé Y, Newman JA, Healy SD (1996). New tests for departures from random behavior in spatial memory experiments. Anim. Learn. Behav..

[63] Bates D (2016). Linear Mixed-Effects using ‘Eigen’ and S4.

[64] Kuznetsova A, Christensen RHB (2017). lmerTest package: Tests in linear mixed effects models. J. Stat. Softw..

[65] R Core Team. *A Language and Environment for Statistical Computing*. (R Foundation for Statistical Computing, 2020).

[66] Warton DI, Lyons M, Stoklosa J, Ives AR (2016). Three points to consider when choosing a LM or GLM test for count data. Methods Ecol. Evol..

[67] Wilson AJ (2018). How should we interpret estimates of individual repeatability?. Evol. Lett..

[68] Stoffel MA, Nakagawa S, Schielzeth H (2017). rptR: repeatability estimation and variance decomposition by generalized linear mixed-effects models. Methods Ecol. Evol..

[69] Hadfield JD (2010). MCMC methods for multi-response generalized linear mixed models: The MCMCglmm R package. J. Stat. Softw..

[70] Houslay TM, Wilson AJ (2017). Avoiding the misuse of BLUP in behavioural ecology. Behav. Ecol..

[71] Kilkenny C, Browne WJ, Cuthill IC, Emerson M, Altman DG (2010). Improving bioscience research reporting: The arrive guidelines for reporting animal research. PLoS Biol..

