# Supplementary material for: Great tits who remember more accurately have difficulty forgetting, but variation is not driven by environmental harshness
Source: Sci Rep. 2021 May 12;11:10083. doi: 10.1038/s41598-021-89125-3 (PMC8114932; doi:10.1038/s41598-021-89125-3)
Supplement: Supplementary file 1 — Supplementary Information 1. [file 41598_2021_89125_MOESM1_ESM.pdf]

Great tits who remember more accurately have difficulty forgetting, but variation is not driven by environmental harshness

Hermer, Ethan*[1], Murphy, Ben[2], Chaine, Alexis S[3,4], Morand-Ferron, Julie[1]

[1]University of Ottawa, Ottawa, ON, Canada.[2]University of Cape Town, Cape Town South Africa.[3]Station d´Ecologie Théorique et Expérimentale du CNRS, Moulis France.[4]Institute for Advanced Studies in Toulouse, Toulouse School of Economics, Toulouse France

Correspondence should be addressed to Ethan Hermer. (email: eherm041@uottawa.ca)

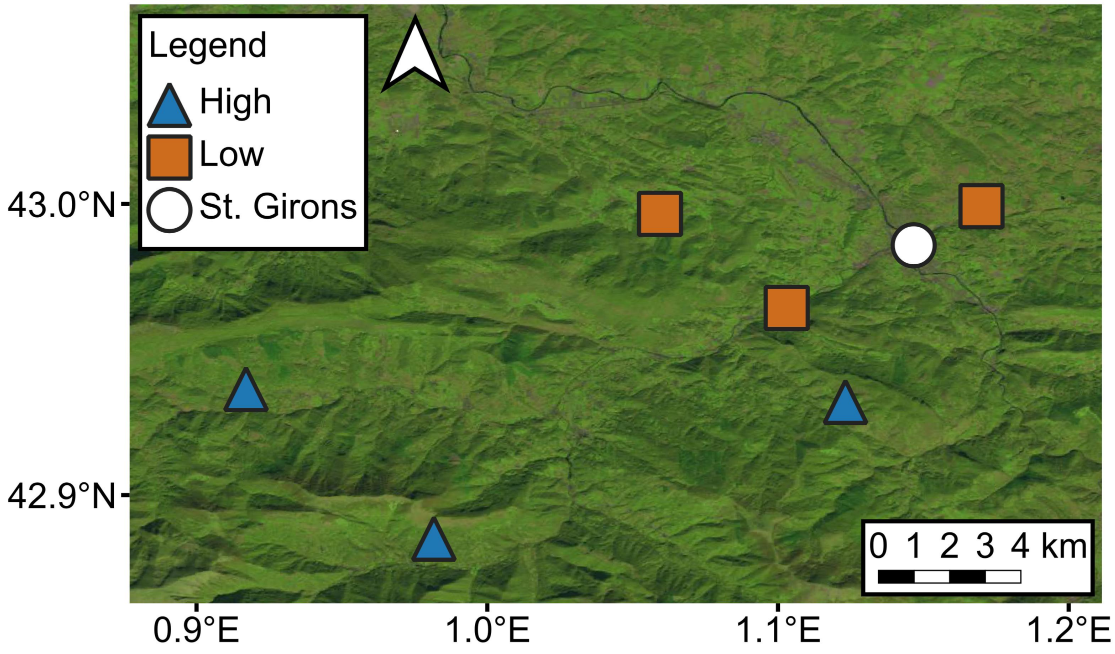

S1. Map of the high-elevation sites (blue triangles; 800-900m) and low-elevation sites (orange squares; 400-500m) near the Experimental and Theoretical Ecology Research Station near St. Girons, France (white circle). Landsat 8 OLI-TIRS Collection 1 scene LC81990302018278LGN00 provided courtesy of U.S. Geological Survey.

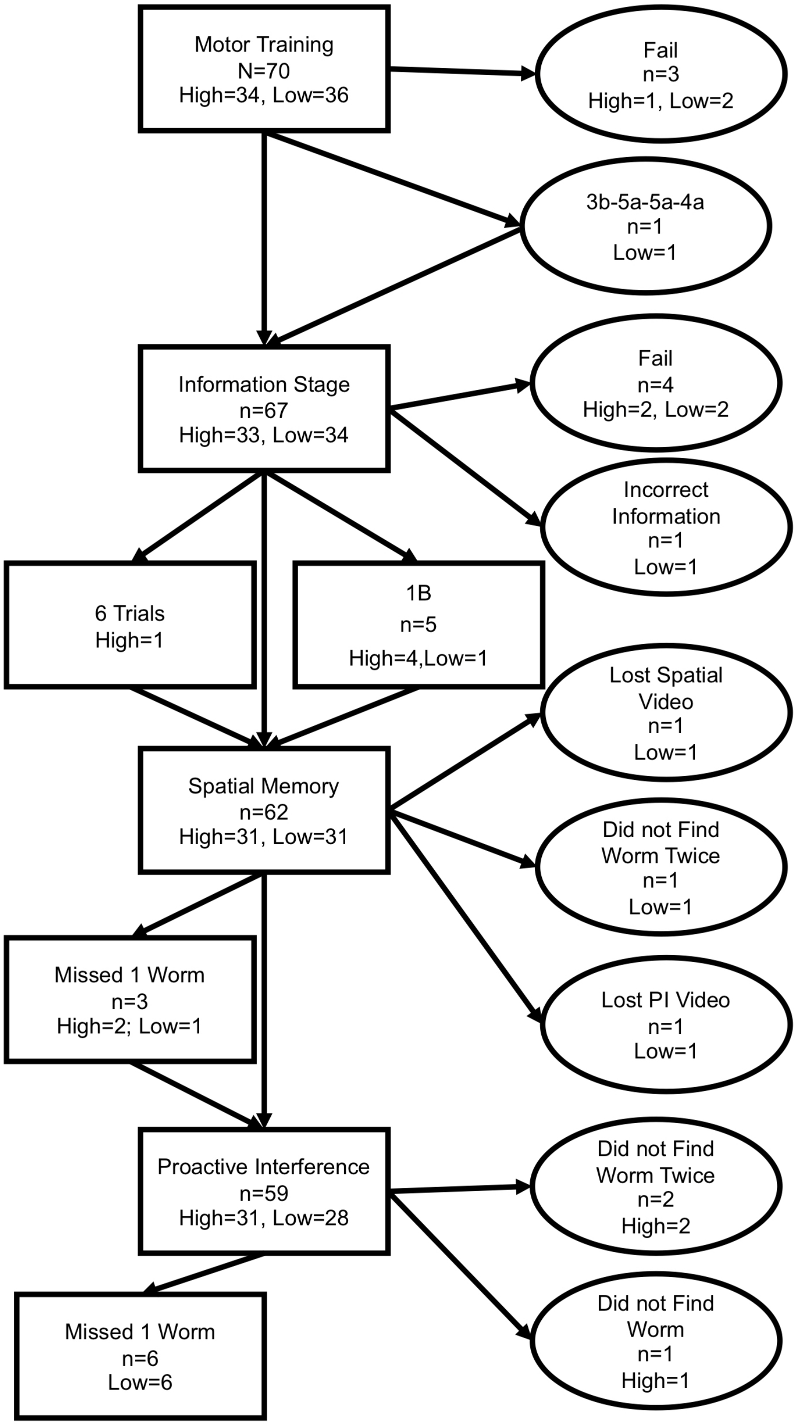

S2. Decision tree for which birds were kept in testing. Squares indicate birds kept in testing; circles denote birds that were removed from testing at that stage.

S3. Individual bird ID's and motor training, spatial memory and proactive interference comments.

Task	Bird ID	Elevation	Comments
Motor Training	1009	High	Did not pass motor training.
Motor Training	117	Low	Did not pass motor training.
Motor Training	923	Low	Did not pass motor training.
Motor Training	123	Low	Went from 3b-5a-5a-4a.
Information Stage	801	High	Did not find worm.
Information Stage	805	High	Did not find worm.
Information Stage	717	Low	Did not find worm.
Information Stage	721	Low	Did not find worm.
Information Stage	713	Low	Given incorrect information during information trial.
Information Stage	811	High	Did not remove worm until second 1b trial.
Information Stage	1001	High	Had 1B for information trial.
Information Stage	809	High	Had 1B for information trial.
Information Stage	715	High	Had 1B for information trial.
Information Stage	917	Low	Had 1B for information trial.
Information Stage	1123	High	Had 1B for information trial.
Spatial Memory	601	Low	Lost Second Day Videos, only has Trials 1-3.
Spatial Memory	1005	High	Did not find worm at Trial 4 but found it during deprivation.
Spatial Memory	513	High	Did not find worm in Trial 2 but found it during deprivation.
Spatial Memory	921	Low	Did not find worm during Trial 1 but found it during deprivation.
Spatial Memory	411	Low	Did not find worm at Trial 2 and Trial 5.
Proactive Interference	119	Low	Lost first day of proactive interference videos.
Proactive Interference	319	High	Did not find worm in the information stage and trial 2.
Proactive Interference	811	High	Did not find worm in the information stage and trial 2.
Proactive Interference	201	High	Does not find worm during Trial 5.
Proactive Interference	123	Low	Does not find worm during the information stage, finds during deprivation.
Proactive Interference	405	Low	Does not find worm during the information stage, finds during deprivation.
Proactive Interference	915	Low	Does not find worm during the information stage, finds during deprivation.
Proactive Interference	919	Low	Does not find worm during the information stage, finds during deprivation.

Proactive Interference	719	Low	Does not find worm during the information stage, finds during deprivation.
Proactive Interference	1211	Low	Does not find worm during Trial 2, finds during deprivation.
Proactive Interference	609	Low	Pulled pompoms early in Trial 3 and Trial 5
Proactive Interference	607	Low	Pulled pompoms early in Trial 3.
Proactive Interference	121	Low	Pulled pompoms early in Trial 3.

S4. Testing schedule for a single batch of birds.

Days 1-6	Day 7	Day 8	Day 9	Day 10	Day 11
Acclimatization	Motor Training	Information Stages and 3 Spatial Memory Trials	4 Spatial Memory Trials	Proactive Interference Information Stage and 3 Proactive Interference Trials	2 Proactive Interference Trials

S5. Predictors of the log transformed number of errors made by high elevation birds (n=31; n=215 trials) across 7 spatial memory trials fitted with a linear mixed effect model with trial, capture order, intertrial interval (min), and site (Cap Sour, Galey, Balacet), sex (male/female), age (juvenile/adult) rewarded side of the tree (left/right), and observer (EH, JH, AR) included as fixed effects. Bird ID was included as a random intercept.

Predictors	Estimate ± SE	F-statistic	*P*
Intercept	2.304 ± 0.773		
Trial	-0.218 ± 0.048	20.912	<.0001
Sex (M)	0.373 ± 0.186	4.018	0.057
Age (Juvenile)	0.105 ± 0.209	0.250	0.622
Capture Order	0.070 ± 0.747	0.009	0.926
Intertrial interval (min)	-0.011 ± 0.048	0.059	0.809
Correct Side (Right)	-0.306 ± 0.176	-3.028	0.096
Observer (EH)	-0.006 ± 1.555	0.044	0.957
Observer (JH)	0.099 ± 0.572		
Site (Cap Sour)	-0.007 ± 0.518	0.367	0.697
Site (Galey)	-0.206 ± 0.286		

S6. Predictors of the log transformed number of errors made by low elevation birds (n=31; n=208 trials) across 7 spatial memory trials fitted with a linear mixed effect model with trial, capture order, intertrial interval (min), and site (Grotte D'Aliou, Montjoie, Aubert), sex (male/female), age (juvenile/adult) rewarded side of the tree (left/right), and observer (EH, JH, AR) included as fixed effects. Bird ID was included as a random intercept.

Predictors	Estimate ± SE	F-statistic	*P*
Intercept	1.821 ± 0.467		
Trial	-0.196 ± 0.046	17.922	<.0001
Sex (Male)	-0.293 ± 0.172	2.898	0.102
Age (Juvenile)	-0.087 ± 0.220	0.158	0.695
Capture Order	0.204 ± 0.129	2.505	0.127
Intertrial Interval (min)	0.052 ± 0.046	1.301	0.256
Correct Side (Right)	-0.015 ± 0.159	0.009	0.926
Observer (EH)	0.800 ± 0.345	2.768	0.084
Observer (JH)	0.574 ± 0.444		
Site (Grotte D'Aliou)	0.119 ± 0.260	0.305	0.740
Site (Montjoie)	0.161 ± 0.221		

S7. Predictors of the log transformed ratio of errors made by high elevation birds (n=31; n=145 trials) across 5 reversal trials fitted with a linear mixed effect model with trial, capture order, intertrial interval (min), and site (Cap Sour, Galey, Balacet), sex (male/female), age (juvenile/adult), and rewarded side of the tree (left/right) included as fixed effects. Bird ID was included as a random intercept.

Predictors	Estimate ± SE	F-statistic	*P*
Intercept	0.675 ± 0.118		
Trial	0.017 ± 0.022	0.5705	0.452
Sex (Male)	-0.045 ± 0.088	0.2667	0.610
Age (Juvenile)	-0.117 ± 0.094	1.5369	0.227
Capture Order	-0.015 ± 0.045	0.1099	0.743
Intertrial Interval	-0.039 ± 0.022	3.1870	0.077
Side (Right)	-0.154 ± 0.083	3.4751	0.075
Site (Cap Sour)	0.051 ± 0.099	0.1407	0.869
Site (Galey)	0.010 ± 0.100		

S8. Predictors of the ratio of errors made by low elevation birds (n=28; n=139 trials) across 5 reversal trials fitted with a linear mixed effect model with trial, capture order, intertrial interval (min), and site (Grotte D'Aliou, Montjoie, Aubert), sex (male/female), age (juvenile/adult) rewarded side of the tree (left/right), and observer (JH, AR) included as fixed effects. Bird ID was included as a random intercept.

Predictors	Estimate ± SE	F-statistic	*P*
Intercept	0.562 ± 0.132		
Trial	0.010 ± 0.026	0.1396	0.709
Sex (Male)	-0.001 ± 0.097	0.0002	0.990
Age (Juvenile)	0.030 ± 0.130	0.0526	0.821
Capture Order	0.060 ± 0.053	1.2703	0.273
Intertrial Interval	0.057 ± 0.026	4.7240	0.032
Side (Right)	-0.018 ± 0.093	0.0369	0.850
Observer (JH)	0.114 ± 0.183	0.3863	0.541
Site (Grotte D'Aliou)	0.097 ± 0.162	0.2058	0.816
Site (Montjoie)	0.031 ± 0.120		

S9. Video of a bird completing a proactive interference trial.
